# Access to Neoadjuvant Pertuzumab for HER2 Positive Breast Cancer in Canada: A Dilemma Increasingly Difficult to Explain

**DOI:** 10.3390/curroncol29120778

**Published:** 2022-12-16

**Authors:** Daniel Rayson, Sonal Gandhi, Anil A. Joy, Christine Brezden-Masley, Karen A. Gelmon, Sandeep Sehdev, David Cescon, Stephen Chia

**Affiliations:** 1 Division of Medical Oncology, Department of Medicine, Queen Elizabeth II Health Sciences Center, Halifax, NS B3H 2Y9, Canada; 2 Division of Medical Oncology/Hematology, Odette Cancer Centre Sunnybrook Health Sciences Centre, Toronto, ON M5M 3J1, Canada; 3 Division of Medical Oncology, Cross Cancer Institute, Edmonton, AB T6G 1Z2, Canada; 4 Division of Medical Oncology, Mount Sinai Hospital, Toronto, ON M5G 1X5, Canada; 5 Division of Medical Oncology, British Columbia Cancer Centre, Vancouver, BC V5Z 4E6, Canada; 6 Department of Medical Oncology, The Ottawa Hospital Cancer Centre, Ottawa, ON K1H 8L6, Canada; 7 Department of Medical Oncology, University Health Network, Toronto, ON M5G 1Z5, Canada

**Keywords:** breast cancer, HER2 positive, neoadjuvant, pertuzumab, access

## Abstract

The addition of pertuzumab to neoadjuvant trastuzumab and chemotherapy for women with early-stage, high-risk, HER2+ breast cancer has been observed to lead to higher pathologic complete response rates (pCR), and improved event-free survival compared to trastuzumab and chemotherapy alone. Based on available data, neoadjuvant pertuzumab is recommended by ESMO, ASCO, and NICE as well as by a Canadian Consensus Guideline Group. We discuss the implications for Canadian patients with HER2+ early breast cancer due to a second and final negative funding decision by the Canadian Agency for Drugs and Technologies in Health (CADTH) related to neoadjuvant pertuzumab. This decision will have adverse impacts for up to 1 in 6 women receiving neoadjuvant therapy for high-risk HER2+ breast cancer, due to suboptimal pCR rates and higher risks of invasive breast cancer recurrent events, resulting in the need for more toxic adjuvant therapy.

## 1. Introduction

Approximately 15–20% of all invasive breast cancers are categorized as HER2 (human epidermal growth factor receptor 2 positive (HER2+). HER2 is an oncogene and when overexpressed or amplified, is an independent adverse prognostic factor and the key molecular driver of this cancer subtype [1,2,3]. An estimated 28,900 cases of invasive breast cancer are expected to be diagnosed in Canada in 2022, resulting in 4335–5780 new patients with HER2+ disease this year [4]. A recent negative funding decision by the Canadian Agency for Drugs and Technologies in Health (CADTH) for neoadjuvant pertuzumab was met with disapproval from the breast cancer community, highlights issues of inequity within Canada, and puts patients at higher risk compared to those living in jurisdictions with publicly funded access to this agent in the pre-operative setting [5,6].

Trastuzumab (Herceptin^®^) was the first systemic therapy developed to specifically target the HER2 protein, demonstrating both progression-free and overall survival (OS) benefit when it was added to chemotherapy for metastatic disease [7]. In the curative-intent adjuvant setting, trastuzumab along with chemotherapy has been utilized since 2005 when results from a series of trials observed improved survival outcomes, compared to chemotherapy alone [8,9]. A second HER2 antibody, pertuzumab (Perjeta^®^), has been incorporated for the first-line treatment of metastatic HER2+ disease since 2015, stemming from a pivotal trial demonstrating a 15-month improvement in median overall survival when it was added to trastuzumab and taxane chemotherapy [10].

Chemotherapy given before definitive surgery, referred to as neoadjuvant therapy (NAT), can down-stage large tumours and allows for earlier treatment administration for breast cancers with a high risk of systemic recurrence such as HER2+ disease. A key observation from neoadjuvant trials is that patients experiencing complete elimination of invasive cancer in both the breast and axillary lymph nodes, termed pathological complete response (pCR), have significantly improved long term disease-free survival (DFS) compared to those with residual cancer [11,12]. This has become increasingly important for high-risk breast cancer subtypes such as HER2+ disease and has led to international efforts to identify treatment regimens and disease characteristics optimizing pCR rates to improve long-term cure. It has also led to pCR being incorporated as a clinically relevant endpoint, along with DFS and OS, in both clinical trials and funding decisions. In Canada, approximately 2200–2900 patients per year with stage II–III HER2+ breast cancer are referred for consideration of neoadjuvant trastuzumab plus chemotherapy which achieves higher pCR rates compared to chemotherapy alone [13].

More recently, dual neoadjuvant HER2-targeted therapy with both pertuzumab and trastuzumab has been observed to improve pCR rates by an additional 16.8% (45.8%; 95% CI 36.1–55.7 versus 29%; 95% CI 20.6–38.5: *p* = 0.0141), compared to trastuzumab alone, leading to a 5% improvement in DFS at 5 years (86%; 95% CI 77–91 versus 81%; 95% CI 71–87) [14]. Based on this data, and the accepted importance of pCR internationally, funding guidelines from Europe (ESMO), the US (ASCO) and the UK (NICE) have all recommended neoadjuvant pertuzumab for patients with high-risk HER2 positive disease [15,16,17,18]. A recent Canadian Consensus Guideline also highlighted the importance of pertuzumab in NAT for HER2+ disease [19]. This guideline also noted the lack of public access which has now become entrenched due to the second negative, and final, funding decision from CADTH [5]. One of the main factors leading to the negative decision was the lack of certainty regarding the impact of pCR on survival outcomes. As well, the impact on DFS was largely discounted, despite several other breast cancer therapies being adopted due to DFS benefit alone (e.g. early data on adjuvant aromatase inhibitors [20], and, as below, adjuvant trastuzumab emtansine).

## 2. Beyond Prognosis and DFS: The Importance of Pathologic Response in Directing Further Therapy

Non-pCR for HER2+ breast cancer has been repeatedly identified as the most significant adverse prognostic factor for both DFS and OS and justified a global clinical trial in an attempt to improve outcomes for this high-risk, non-pCR, HER2+ patient population. As such, in the pivotal KATHERINE trial, patients with residual HER2 positive disease following neoadjuvant chemotherapy plus trastuzumab were randomized to continuing trastuzumab or were switched to the HER2 drug-antibody conjugate trastuzumab emtansine (T-DM1), for 14 cycles to complete 1 year of treatment. Invasive disease-free survival [IDFS] was improved at 3 years (88.3% in the T-DM1 arm versus 77% with trastuzumab alone, HR 0.5; *p* < 0.001) favoring the switch [21]. Based on this data, T-DM1 was approved by CADTH for the adjuvant treatment of HER2+ breast cancer patients not achieving a pCR following neoadjuvant trastuzumab and chemotherapy [22]. Although there has not, as of yet, been a statistically significant improvement in OS from TDM1 therapy, it has become a standard of care in this clinical situation.

Importantly, adjuvant T-DM1 for 14 cycles is both more toxic and costly than neoadjuvant pertuzumab for 4–6 cycles, with common adverse events including leukopenia, thrombocytopenia, fatigue, peripheral neuropathy, and hepatic transaminitis leading to 17% of patients on the clinical trial discontinuing T-DM1 early [21]. This compares unfavorably to neoadjuvant pertuzumab, which adds little to no excess toxicity when added to current NAT. For 1 in 6 patients, incorporation of neoadjuvant pertuzumab could improve the chance of pCR and prognosis, while sparing them the more intensive monitoring requirements and toxicities of additional T-DM1.

The cost analysis for neoadjuvant pertuzumab performed by CADTH did not account for the cost savings accrued through avoidance of TDM-1 for those additional patients achieving a pCR, nor the cost savings accrued by avoidance of breast cancer recurrent events. As well, it assumed a full year of pertuzumab regardless of pCR status, whereas neoadjuvant pertuzumab followed by adjuvant trastuzumab alone for those patients with a pCR, as was done in the pivotal NeoSphere trial, could be a consideration [11]. Adjuvant pertuzumab also received a negative CADTH recommendation based on early results from the APHINITY trial, although with longer follow-up (median 74 months) the node positive patient population experienced a 5% improvement in invasive disease-free survival (IDFS) (88% vs 83%; HR 0.72, 95% CI: 0.59–0.87) [23]. Canadian patients with high-risk HER2+ breast cancer, therefore, remain at a significant disadvantage compared to patients in other jurisdictions with public access to optimal HER2-based therapy.

The negative funding decision for neoadjuvant pertuzumab means that roughly 1 in 6 women with HER2+ breast cancer receiving NAT will need more toxic and costly systemic therapy after surgery, rather than receiving optimal treatment pre-operatively. Additionally, evolving treatment algorithms integrate the combined efficacy of neoadjuvant trastuzumab and pertuzumab to allow for treatment de-escalation (e.g. sparing anthracyclines to minimize cardiac risk), without compromising patient outcomes [24]. This decision will, therefore, further impact access to novel therapeutic strategies in development for the majority of Canadian patients with HER2+ breast cancer.

Within Canada, the Institut National D’excellence en Sante et en Services Sociaux (INESSS) in Quebec issued a positive funding recommendation largely driven by acknowledging pCR as the most important primary endpoint of NAT for HER2 positive breast cancer, based on the identical data reviewed by CADTH [25]. Arriving at different conclusions based on interpretation of the relevance of pCR is a unique Canadian dilemma impacting patient care. Outside Quebec, patients only have access to this therapy through private insurance or personal payment and depending on cancer centre policies, a patient with the same disease may be receiving this more effective therapy while sitting next to one who had access denied.

Without equitable access to neoadjuvant pertuzumab, Canadian patients with HER2+ breast cancer are being disadvantaged in multiple domains with implications for clinical outcomes, toxicity, and quality of life. We believe the improvement in pCR attained from neoadjuvant pertuzumab is an important endpoint which improves the prognosis of 1 in 6 patients, and saves them from needing additional, more toxic therapy in the form of adjuvant TDM1. If modelled with the option of neoadjuvant pertuzumab and less TDM1 use, along with financial savings from fewer subsequent breast cancer disease-related events, this treatment strategy could also lead to cost savings for the health care system. Neoadjuvant pertuzumab is an evolving global standard in OECD (Organization for Economic Co-operation and Development) nations, and this decision likely disadvantages many of our patients by limiting access to newer treatment approaches that rely on this treatment approach. As a group of medical oncologists treating breast cancer, it is no longer possible to explain the rationale and justification for the CADTH funding decision to our patients, nor to those looking to us for expert guidance on optimal curative-intent therapies for HER2 positive breast cancer.
